# The meiotic topoisomerase VI B subunit (MTOPVIB) is essential for meiotic DNA double-strand break formation in barley (*Hordeum vulgare* L.)

**DOI:** 10.1007/s00497-022-00444-5

**Published:** 2022-06-29

**Authors:** Stefan Steckenborn, Maria Cuacos, Mohammad A. Ayoub, Chao Feng, Veit Schubert, Iris Hoffie, Götz Hensel, Jochen Kumlehn, Stefan Heckmann

**Affiliations:** Leibniz Institute of Plant Genetics and Crop Plant Research (IPK) OT Gatersleben, Corrensstrasse 3, 06466 Seeland, Germany

**Keywords:** Barley, Meiosis, Meiotic recombination, MTOPVIB, Meiotic DSB, Meiotic spindle

## Abstract

**Key message:**

In barley (*Hordeum vulgare*), *MTOPVIB* is critical for meiotic DSB and accompanied SC and CO formation while dispensable for meiotic bipolar spindle formation.

**Abstract:**

Homologous recombination during meiosis assures genetic variation in offspring. Programmed meiotic DNA double-strand breaks (DSBs) are repaired as crossover (CO) or non-crossover (NCO) during meiotic recombination. The meiotic topoisomerase VI (TopoVI) B subunit (MTOPVIB) plays an essential role in meiotic DSB formation critical for CO-recombination. More recently MTOPVIB has been also shown to play a role in meiotic bipolar spindle formation in rice and maize. Here, we describe a meiotic DSB-defective mutant in barley (*Hordeum vulgare* L.). CRISPR-associated 9 (Cas9) endonuclease-generated *mtopVIB* plants show complete sterility due to the absence of meiotic DSB, synaptonemal complex (SC), and CO formation leading to the occurrence of univalents and their unbalanced segregation into aneuploid gametes. In *HvmtopVIB* plants, we also frequently found the bi-orientation of sister kinetochores in univalents during metaphase I and the precocious separation of sister chromatids during anaphase I. Moreover, the near absence of polyads after meiosis II, suggests that despite being critical for meiotic DSB formation in barley, *MTOPVIB* seems not to be strictly required for meiotic bipolar spindle formation.

**Supplementary Information:**

The online version contains supplementary material available at 10.1007/s00497-022-00444-5.

## Introduction

Meiosis in sexually reproducing organisms assures genetic variation through homologous recombination (HR) (Hunter, 2015; Mercier et al., 2015). Meiotic HR starts with DNA double-strand break (DSB) formation catalyzed by the topoisomerase-like protein SPO11 (Bergerat et al., 1997; Keeney et al., 1997). Meiotic DSBs repaired using the homologous chromosome as template result either in crossovers (COs; reciprocal genetic exchange between homologous chromosomes) or non-crossovers (NCOs; only rather short stretches of DNA are copied to the broken chromosome) (Hunter, 2015; Mercier et al., 2015; Wang and Copenhaver, 2018). Typically, only a minor fraction of meiotic DSBs becomes repaired into COs in plants. Meiotic DSBs are generated in excess with more than 90% of plant DSBs being resolved as NCO or being repaired by using the sister chromatid as a template (Mercier et al., 2015). The remaining DSBs can mature into two types of COs in plants: Interference-sensitive class I COs that are farther apart along the chromosome than expected by chance (CO interference; (Berchowitz and Copenhaver, 2010)) and depend on the activity of a group of proteins called ZMM proteins (named after the budding yeast proteins Zip1-4, Mer3, Msh4 and Msh5 (Pyatnitskaya et al., 2019)) across organisms including different plants (e.g., Chelysheva et al., 2012; Shen et al., 2012; Desjardins et al., 2020) as well as ZMM-independent COs that are interference-insensitive (class II) and depend in part on MUS81 or FANCD2 (Berchowitz et al., 2007; Higgins et al., 2008; Kurzbauer et al., 2018). In plants, the majority of CO are ZMM-dependent class I COs (Mercier et al., 2015; Wang and Copenhaver, 2018).

While in many species a single *SPO11* gene is present (e.g., mammals and fungi) with two functionally distinct splice variants as in mice and humans (Bellani et al., 2010; Kauppi et al., 2011), three *SPO11* genes are found in *A. thaliana*. Two of them, *SPO11-1* and *SPO11-2*, become differentially spliced (Sprink and Hartung, 2014, 2021) and are required for meiotic DSB formation (Grelon et al., 2001; Stacey et al., 2006; Hartung et al., 2007), while the third, *SPO11-3*, is critical for somatic development (Hartung et al., 2002; Sugimoto-Shirasu et al., 2002; Yin et al., 2002) but not required for meiotic DSB formation. In rice, among five *SPO11* genes at least *SPO11-1* and *SPO11-2* are involved in meiotic DSB formation (Yu et al., 2010; An et al., 2011; Fayos et al., 2020). In wheat, *SPO11-1* and *SPO11-2* (Benyahya et al., 2020; Da Ines et al., 2020), and in maize, *SPO11-1* (Ku et al., 2020) are critical for meiotic DSB formation. In *A. thaliana* (L.) Heynh., SPO11-1 and SPO11-2 form a heterotetramer with two copies of the distant archaeal topoisomerase VI (TopoVI) B subunit homolog MTOPVIB (Vrielynck et al., 2016) constituting the meiotic TopoVI-like complex (core catalytic meiotic DSB induction complex). MTOPVIB contains four protein motifs that are conserved among flowering plants, named B1, B2, B3, and B4 from N- to C-terminus. The B1 and B2 motifs are part of the GHKL domain, necessary for ATP binding. The function of the B3 motif is currently unclear. The B4 motif is part of the Transducer domain mediating the interaction with SPO11-1 and SPO11-2 (Vrielynck et al., 2016). MTOPVIB is critical for meiotic DSB induction in *A. thaliana*, rice, maize, and mice (Fu et al., 2016; Robert et al., 2016a; Vrielynck et al., 2016; Xue et al., 2016; Jing et al., 2020). Further accessory proteins are also required for DSB formation in plants (e.g., PRD1-3 or DFO in *A. thaliana*; (De Muyt et al., 2007, De Muyt et al., 2009, Zhang et al., 2012, Vrielynck et al., 2021)). Functional impairment of any of the core DSB induction complex components or any accessory factor results in abolition of meiotic DSB formation, leading to a severe fertility reduction due to the absence of meiotic HR and accompanied unbalanced gamete formation.

In addition to its role in meiotic DSB formation, MTOPVIB, initially in rice (Xue et al., 2019) but also in maize (Jing et al., 2020), has been implicated in being critical for bipolar spindle formation at metaphase I, which is independent of its role in DSB formation. In *mtopVIB,* a high frequency of polyads (up to octads) is found in rice (~ 86% Xue et al., 2019), maize (~ 63% Jing et al., 2020), and in *A. thaliana* (~ 70% Tang et al., 2017). Moreover, at metaphase I, sister kinetochores in rice *mtopVIB* univalents are typically mono-oriented while in univalents in other rice mutants defective for meiotic DSB formation, frequent bi-orientation of sister kinetochores is found (Xue et al., 2019). Hence, likely the bi-orientation of kinetochores in (at least some) univalents at metaphase I is critical for the bipolar spindle organization in case of the absence of meiotic DSBs in an MTOPVIB-dependent manner (Xue et al., 2019).

Here we describe the function of *MTOPVIB* in barley. Cas9 endonuclease-generated *mtopVIB* plants are entirely sterile due to defective meiosis. In *HvmtopVIB*, no meiotic DSBs form, synaptonemal complex (SC) formation is abolished, and the absence of CO formation leads to univalents that undergo unbalanced segregation into aneuploid gametes. Moreover, frequent bi-orientation of sister kinetochores during metaphase I, precocious separation of sister chromatids during anaphase I, and rare polyad formation were found in *mtopVIB* lines. Hence, barley MTOPVIB seems not to be required for bipolar spindle formation despite its role in meiotic DSB formation.

## Materials and methods

### Plant material, growing conditions, and crossing

Grains of the wild-type (WT) barley (*Hordeum vulgare* L.) cultivar Golden Promise and *mtopVIB* mutants were germinated on wet filter paper in a Petri dish. After seven days, plantlets were transferred to soil pots and grown in a greenhouse at 18/15 °C and under 16 h light/8 h darkness conditions with a relative humidity of 60–70%.

Plant crossings were done as described (Ahn et al., 2020) by using five-to-seven-week-old plants. Spikes for emasculation were selected based on the size and appearance of emerging awns. All three immature anthers from each spikelet along the whole spike were removed. Two days after emasculation, 2–3 freshly collected mature anthers were introduced into each of the emasculated spikelets. The developing caryopses became visible after 10 to 12 days in cases of a successful pollination. Mature spikes were collected to estimate the number of generated grains per cross.

### Resequencing of *HvMTOPVIB* and expression analysis

Total RNA was isolated from 100 mg of barley anthers using Trizol (Invitrogen). One µg of RNA was employed for cDNA synthesis using the Invitrogen FirstStrand cDNA Synthesis Using SuperScript II RT kit following manufacturer’s instructions. cDNA samples were used for PCR amplification using the NEB Phusion High Fidelity PCR Kit and Sanger resequencing of the coding sequence of barley *MTOPVIB*. For primer sequences see Online resource 1.

### Selection of Cas9/gRNA target motifs and Cas9-mediated in vitro digestion

The coding sequence of *HvMTOPVIB* was cloned using the CloneJET PCR cloning kit following the manufacturer's instructions (ThermoFisher). The resulting plasmid DNA was linearized with XbaI. Cas9/gRNA target motifs were selected within the coding sequence of *HvMTOPVIB* according to criteria previously described (Kumlehn et al., 2018). Three accordingly designed *HvMTOPVIB* crRNAs (single-strand RNAs that contain the gRNA target sequence) for exon 1 (gRNA#1: GAGCTTCCGGTGGGGGGAGG), exon 2 (gRNA#2: GGATGTCGGAGTCGCAGTGC), and exon 6 (gRNA#3: GACTTCATATTATGGCTGGT) were hybridized to the tracrRNA by dissolving 2 nmol each in Nuclease-Free Duplex Buffer (Integrated DNA Technologies, IDT). To generate the ribonucleoprotein (RNP) complex (Cas9 endonuclease combined with the hybridized gRNAs), 1 µl of the RNA mix was combined with 1 µl of Cas9 solution (1 µM Cas9 endonuclease purchased from IDT, 1 × PBS, and 50% glycerol) in 1 × PBS. The digestion was performed by mixing the RNP complex solution with 200 ng of linearized plasmid DNA followed by incubation at 37 °C for 1 h and analyzed by running the samples in an agarose gel.

### Construction of transformation vectors

Using the CasCADE vector system (Hoffie et al., 2022), target-specific sequences (see Online resource 1) were integrated downstream of a wheat *TaU6* promoter into generic gRNA modules which were then used to create guide RNA assembly vector pGH577. Then, the final assembly vector pGH584 was produced including a maize codon-optimized *xcas9* (Hu et al., 2018) driven by the maize *Polyubiquitin 1* promoter along with the 5’-UTR including intron 1. From pGH584, the SfiI-fragment containing gRNA and *xcas9* expression units were transferred to the compatible generic binary vector 271p6i-2 × 35 s-TE9 (DNA Cloning Service, Hamburg, Germany) to create pGH615.

### Plant transformation

The generation of transgenic barley plants was done as previously described (Hensel et al., 2008; Marthe et al., 2015). In brief, immature embryos were dissected from surface-sterilized caryopses, co-cultivated upon inoculation with the hypervirulent *Agrobacterium tumefaciens* strain AGL1 (Lazo et al., 1991) harboring plasmid pGH615, which was followed by callus induction and plant regeneration under selective conditions using Timentin to remove *Agrobacterium* and hygromycin to ensure preferential development of transgenic cells and tissue. When the plantlets had developed roots, they were transferred into soil.

### Identification of mutations within *HvMTOPVIB*

The genomic regions addressed for targeted mutagenesis (a fragment of 628 bp spanning the target motifs of gRNA#1 and gRNA#2 as well as a fragment of 411 bp harboring the cognate motif of gRNA#3) were PCR-amplified from flag leaf genomic DNA of primary transformants (T_0_) and Sanger-sequenced. For primer sequences see Online resource 1. In segregating T_1_ families (35 siblings per T_0_ parent), the target regions were PCR-amplified and resulting agarose gel-purified amplicons were sent to Sanger-sequencing. T-DNA-free individuals were PCR-selected using primers for *cas9* and *hpt*. For primer sequences see Online resource 1.

### Plant genotyping

*HvmtopVIB* is associated with a disruption of a BtsI restriction site within *HvMTOPVIB*, which facilitates genotyping. After PCR amplification of the previously described 628-bp PCR fragment spanning the target motifs of gRNA#1 and gRNA#2 (for primer sequences see Online resource 1), resulting amplicons were purified and used in a BtsI restriction digest. PCR amplicons from the *HvMTOPVIB* WT allele are digested by Btsl (287, 252, and 90 bp fragments) while PCR amplicons from plants carrying a mutant *mtopVIB* allele are only partially digested (343 and 287 bp fragments) enabling plant genotyping.

### Male meiotic chromosome preparations

Spikes were fixed in an ice-cold 3:1 (75% ethanol, 25% acetic acid) solution for at least 24 h. One anther per spikelet was used to determine the meiotic stage by squashing it on a microscopic slide in a drop of acetocarmine (MORPHISTO) and evaluation under a light microscope. The remaining two anthers of selected spikelets were either used immediately or stored in freshly-prepared 3:1 solution. Male meiotic chromosomes were prepared by squashing as described (Li et al., 2018). Anthers were disrupted with the help of forceps in a drop of acetocarmine solution, the preparation was heated by passing the slide 2–3 times above a flame (the acetocarmine should not boil), and a glass coverslip was placed on top pressing gently and firmly with a thumb between filter paper. At this stage, male meiotic chromosomes can be visualized under a light microscope. Staining can be enhanced by adding additional acetocarmine solution at the edges of the coverslip, allowing it to penetrate by diffusion, and by incubating slides in a moist chamber at 4 °C overnight. This also enables staining of the meiotic spindle. To counterstain chromosomes with DAPI, slides were frozen in liquid nitrogen, the coverslips removed with the help of a razor blade, and immediately transferred to an ethanol series (70, 85, 100%) 2 min each for dehydration. After airdrying, slides were counterstained with DAPI in Vectashield mounting media (1.5 µg/ml, Vector Laboratories).

### Immunohistochemistry

One fresh anther per spikelet was used to determine meiotic stages as indicated above. The remaining two anthers of selected spikelets were used for chromosome spreading as described (Armstrong et al., 2009; Cuacos et al., 2021) with minor modifications. Two anthers per slide were digested in 25 µl of enzyme mix for 8 min at 37 °C in a moist chamber, disrupting the material with a brass rod after the first 4 min. Spreading was done with 17 µl of 1.5% Lipsol solution and fixation with 17 µl of 4% paraformaldehyde. Primary antibodies were incubated overnight at 4 °C, and secondary antibodies for 1 h at 37 °C. The following primary antibodies and dilutions were used: anti-HvHEI10 (guinea pig (Desjardins et al., 2020), 1:200), anti-OsγH2AX (rabbit (Miao et al., 2013), 1:200), anti-HvASY1 (guinea pig, 1:500), anti-HvZYP1 (rabbit, 1:500), and anti-grass CENH3 (rabbit (Sanei et al., 2011), 1:300). Secondary antibodies and dilutions used were: goat anti-guinea pig Alexa488 (Invitrogen), goat anti-guinea pig Alexa594 (Invitrogen), donkey anti-rabbit Alexa488 (Jackson Immunology Research), and donkey anti-rabbit Alexa594 (Invitrogen), all at 1:500.

### Fluorescence in situ hybridization

All steps were performed at room temperature unless otherwise indicated. Slides with DAPI-stained male meiotic chromosomes prepared as described above were rinsed in 2xSSC to let coverslips fall, followed by an alcohol series (70, 85, 100%) and air-dried. Next, slides were washed twice in 2xSSC for 5 min, treated with 45% acetic acid for 10 min, washed in 2xSSC for 10 min, treated with 0.1% pepsin in 0.01 N HCl for 10 min at 37 °C, rinsed twice with 2xSSC for 5 min, fixed in 4% formaldehyde (5 ml 37% formaldehyde + 42 ml 2xSSC) for 10 min followed by three washes in 2xSSC of 5 min each. Afterward, slides were dehydrated in ethanol (70, 85, 100%, 2 min each) and air-dried for 1 h. 20 µl hybridization mix (10 µl deionized formamide, 5 µl 4 × buffer (4 × buffer: 80 µl 20xSSC, 8 µl 1 M Tris–HCL pH 8.0, 1.6 µl 0.5 M EDTA, 99.2 µl double-distilled water (ddH2O)), 2 µl ddH2O and 3 µl probe) was added to each slide under a 22 × 22 mm coverslip, slides were sealed with rubber cement (Fixogum, Marabu), denatured at 80 °C for 2 min on a hot plate and immediately transferred to a moist chamber for incubation overnight at 37 °C. On the next day, coverslips were carefully removed, slides were washed in 2xSSC for 20 min at 58 °C, transferred to 2xSSC at room temperature, and dehydrated in 70, 85, 100% ethanol, 2 min each. After airdrying, slides were counterstained with DAPI in Vectashield mounting media and evaluated under a fluorescence microscope.

Probes utilized were: 5S (pCT4.2, Campell et al., 1992) and 45S (pTa71, Gerlach and Bedbrook, 1979) rDNA as well as HvT01 (Rey et al., 2018). FISH probes were labeled by nick translation with Texas Red and Atto488 (NT labeling kits, Jena Biosciences).

### Microscopy

Images were acquired with a Nikon Eclipse Ni-E fluorescence microscope equipped with a Nikon DS-Qi2 camera and NIS-Elements-AR version 4.60 software (Nikon, Tokyo, Japan). Images were processed with GIMP 2.10 (www.gimp.org). To detect the ultrastructural chromatin organization of meiocytes at a resolution of ~ 120 nm (super-resolution achieved with a 488 nm laser excitation) spatial structured illumination microscopy (3D-SIM) was performed with a 63 × /1.4 Oil Plan-Apochromat objective of an ElyraPS.1 microscope system and the software ZENBlack (Carl Zeiss GmbH). Images were captured separately for each fluorochrome using the 561, 488, and 405 nm laser lines for excitation and appropriate emission filters (Weisshart et al., 2016; Kubalová et al., 2021). Maximum intensity projections of whole cells were calculated via the ZENBlack software.

### Yeast two-hybrid assays

To fuse *H*v*SPO11-1, HvSPO11-2, HvMTOPVIB* or *HvmtopVIB* to the Gal4activation domain (AD, prey vector) or the Gal4DNA-binding domain (BD, bait vector), each CDS was cloned into pGADT7 and pGBKT7 vectors. For primer sequences see Online resource 1. Different combinations of prey and bait plasmids were co-transformed into the yeast strain Y2HGold. Handling of yeast transformation and selection assays were performed according to the manufacturer’s instructions (Takara, #630,489). Transformed yeast cells were grown on different selective media: TDO (Minimal Media Triple Dropouts, SD/–His/–Leu/–Trp, #630,419), QDO (Minimal Media Quadruple Dropouts, SD/–Ade/–His/–Leu/–Trp, #630,428) and DDO (Minimal Media Double Dropouts, SD/–Leu/–Trp, #630,417).

### Antibody production

To produce anti-HvZYP1 antibodies, a synthetic peptide (HPANIGELFSEGSLNPYADD) corresponding to aa 839–858 of *H. vulgare* ZYP1 was used for immunization of rabbits by LifeTein. Rabbit anti-HvZYP1 was affinity-purified against the synthetic peptide by LifeTein.

To produce anti-HvASY1, the complete *HvASY1* CDS was used to produce recombinant HvASY1 proteins by Biomatik. Recombinant HvASY1 proteins were used for the immunization of guinea pigs by Davids Biotechnologie GmbH.

## Results

### Identification of barley *MTOPVIB*

**Fig. 1:**
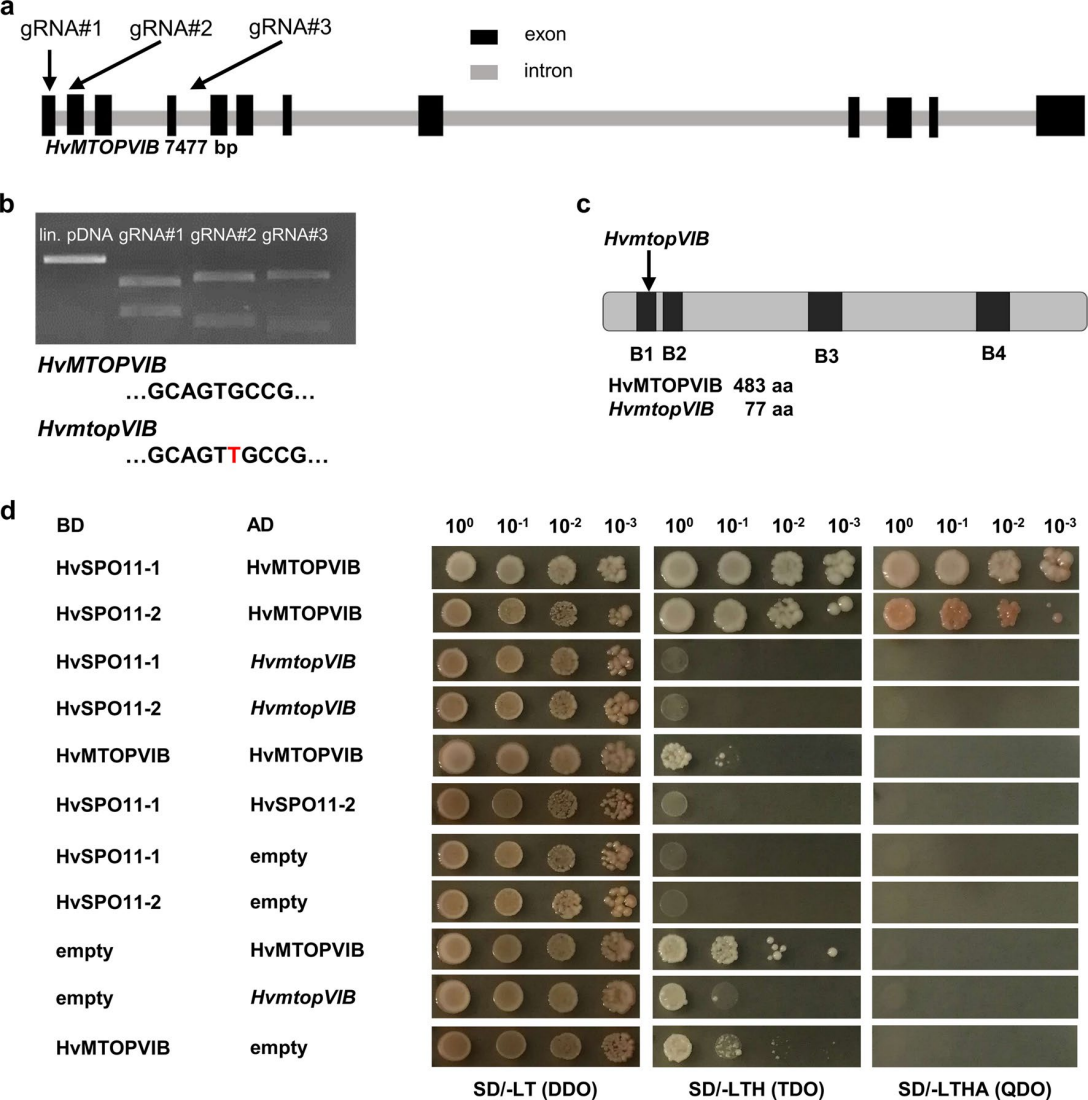
Identification of the *HvMTOPVIB* gene and isolation of a *HvmtopVIB* mutant. **a** *HvMTOPVIB* schematic gene structure depicting exons and introns. **a, b** Three selected gRNAs targeting exon one (gRNA#1), two (gRNA#2), or six (gRNA#3) of *HvMTOPVIB* are active in vitro. *HvmtopVIB* mutation (T insertion present in *HvmtopVIB* as compared with the *HvMTOPVIB* CDS) recovered in independent transgenic families and plants. **c** Schematic protein model of HvMTOPVIB depicting *HvmtopVIB* and all four conserved MTOPVIB domains (B1, B2, B3, and B4). **d** *HvMTOPVIB* while not *HvmtopVIB* interacts with *HvSPO11-1* and *HvSPO11-2* in “yeast two-hybrid assays”. No interaction was found between *HvSPO11-1* and *HvSPO11-2.* Interactions were verified by the growth of yeast cells on different selective media: TDO (Minimal Media Triple Dropouts, SD/–His/–Leu/–Trp), QDO (Minimal Media Quadruple Dropouts, SD/–Ade/–His/–Leu/–Trp) and DDO (Minimal Media Double Dropouts, SD/–Leu/–Trp). BD, bait vector; AD, prey vector

To identify potential candidates involved in meiotic DSB induction in the cereal crop barley, the barley reference genome was initially queried for putative *SPO11* and *MTOPVIB* homologs (The International Barley Genome Sequencing Consortium 2012). In the latest MorexV3 genome reference (Mascher et al., 2021) three putative *SPO11* candidates (*SPO11-1*—HORVU.MOREX.r3.5HG0511100.1, *SPO11-2*—HORVU.MOREX.r3.7HG0699350.1, *SPO11-3*—HORVU.MOREX.r3.4HG0385890.1) and a single *MTOPVIB* candidate (HORVU.MOREX.r3.7HG0726050) were found. We focused on the single copy *MTOPVIB* candidate. The *MTOPVIB* candidate locus represents a putative ORF of 7477 bp predicted to encode for twelve exons and two different CDS variants of 1449 and 1452 bp (The International Barley Genome Sequencing Consortium 2012) (Fig. 1a). While the first predicted CDS variant results in a 484 aa protein containing all four conserved MTOPVIB domains (B1, B2, B3, and B4), the second predicted variant results in a 354 aa protein lacking part of the Transducer domain. In cDNA samples from anthers of cv. Golden Promise, only the first but not the second CDS variant was found at detectable levels, suggesting abundant expression of the first variant in reproductive tissues.

### Generation of *HvmtopVIB* plants by Cas9-triggered mutagenesis

To functionally dissect the role of meiotic DSB induction for CO formation in barley, the single copy candidate *MTOPVIB* was selected as the target for RNA-guided Cas9 to isolate DSB-defective plants. *MTOPVIB* was initially identified in the Morex reference genome. Typically, cv. Golden Promise is employed for stable genetic transformation including the application of Cas endonuclease technology (Koeppel et al., 2019). The conservation of the *MTOPVIB* coding sequence between cvs. Morex and Golden Promise was confirmed based on sequencing of the *MTOPVIB* coding sequence from respective anther cDNA samples. Three Cas9-compatible gRNAs addressing either exon one (gRNA#1), two (gRNA#2), or six (gRNA#3) of *HvMTOPVIB* were selected and their target-specific activity was confirmed in vitro (Fig. 1a, b). These three gRNAs were independently placed under the control of a wheat *U6* promoter in a binary vector carrying *cas9* under control of the maize *Ubiquitin 1* promoter and *hpt* as plant selection marker, and used for stable genetic transformation of cv. Golden Promise via *Agrobacterium*-mediated DNA transfer to immature embryo explants. Among 18 regenerated T_0_ plants, Sanger sequencing of Cas9/gRNA target regions revealed potential mutations in four independent plants (named E1, E7, E9, and E12) at the target motifs in exon one and exon two. In 35 progeny of each E1, E7, E9, and E12 T_0_ plants, heritable edits were only recovered in the gRNA#2-addressed target motif (in exon two) within the T_1_ families of E7 and E9. Among these, three *cas9*-free T_1_ plants with a heterozygous one-base pair thymidine insertion 189 bp downstream of the ATG of *HvMTOPVIB* (termed *HvmtopVIB*) were identified (Fig. 1b, c). The presence of this mutation (at position + 103 relative to the ATG of the CDS; exon 2) was confirmed based on sequencing of anther cDNA samples. *HvmtopVIB* leads to a predicted frameshift in the *HvMTOPVIB* reading frame from the middle of the B1 protein motif, which results in a nonsense aa sequence and a premature STOP codon. The predicted aberrant truncated protein is 77 aa long and lacks all conserved functional domains of MTOPVIB. To confirm whether the insertion had an impact on *HvMTOPVIB* function, the *HvMTOPVIB* cDNA of *HvmtopVIB* and WT plants was used in Y2H experiments together with the CDS of *HvSPO11-1* and *HvSPO11-2* (Fig. 1d). Only yeast cells transformed with *HvMTOPVIB* and *HvSPO11-1* or *HvSPO11-2* grew on selective media suggesting an interaction of *HvMTOPVIB* with *HvSPO11-1* and *HvSPO11-2*. Notably, the one-base pair insertion in *HvmtopVIB* disrupts the interaction of HvMTOPVIB with HvSPO11-1 and HvSPO11-2, suggesting that the mutation recovered in independent transgenic families and plants results in an aberrant truncated protein disrupting the function of HvMTOPVIB.

### *HvmtopVIB* plants are sterile despite normal vegetative growth

**Fig. 2:**
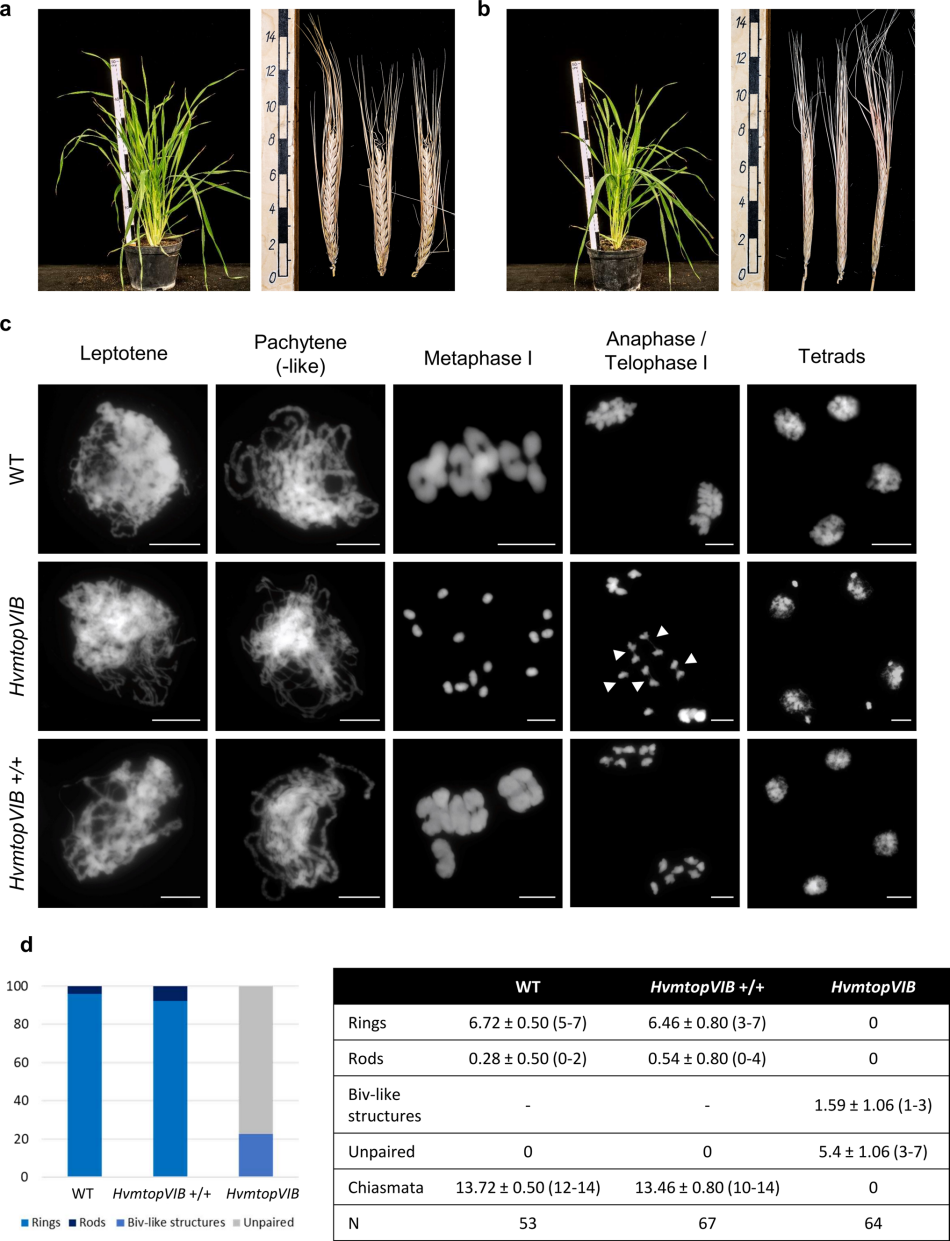
Phenotype of *HvmtopVIB* barley plants: Vegetative growth and development similar to WT, but complete sterility due to meiotic defects. **a, b** Six-weeks old plants and mature spikes from WT (Golden Promise) (**a**) and *HvmtopVIB* (**b**). No grains formed in *HvmtopVIB*. **c** Male meiotic chromosome analysis in WT, *HvmtopVIB,* and *HvmtopVIB* + / + . Compared with WT and segregating *HvmtopVIB* + / + plants, in *HvmtopVIB* the absence of thick chromosomes indicates no synapsis during pachytene and the presence of 14 univalents indicates the absence of COs at metaphase I. During anaphase I, random segregation of univalents to opposite poles or separation of sister chromatids (arrowheads) finally leading to the formation of unbalanced tetrads and micronuclei in *HvmtopVIB*. DNA stained with DAPI and shown in grey. **d** Quantification of bivalent/univalent formation in WT, *HvmtopVIB* + / + and *HvmtopVIB*. Left: among all bivalents, percentage of ring and rod bivalents (in WT and *HvmtopVIB* + / +) and bivalent-like (“biv-like”) structures and “unpaired” bivalents (i.e., pairs of univalents) in *HvmtopVIB*. Right: absolute values. Bars represent 10 µm

Vegetative growth and development of homo- and heterozygous *HvmtopVIB* plants were similar to WT plants. However, homozygous *HvmtopVIB* plants were completely sterile, i.e., spikes were devoid of grains, while WT and heterozygous *HvmtopVIB* plants were fertile (Fig. 2a, b). To address whether also female fertility was disturbed, *HvmtopVIB* plants as well as segregating WT plants were pollinated with WT pollen. While pollinated WT plants formed grains, in the case of *HvmtopVIB* plants, no grains were recovered whatsoever, suggesting that both male and female fertility were severely impaired.

### Absence of bivalent and CO formation as well as unbalanced chromosome segregation and gametes in *HvmtopVIB* mutants

**Fig. 3:**
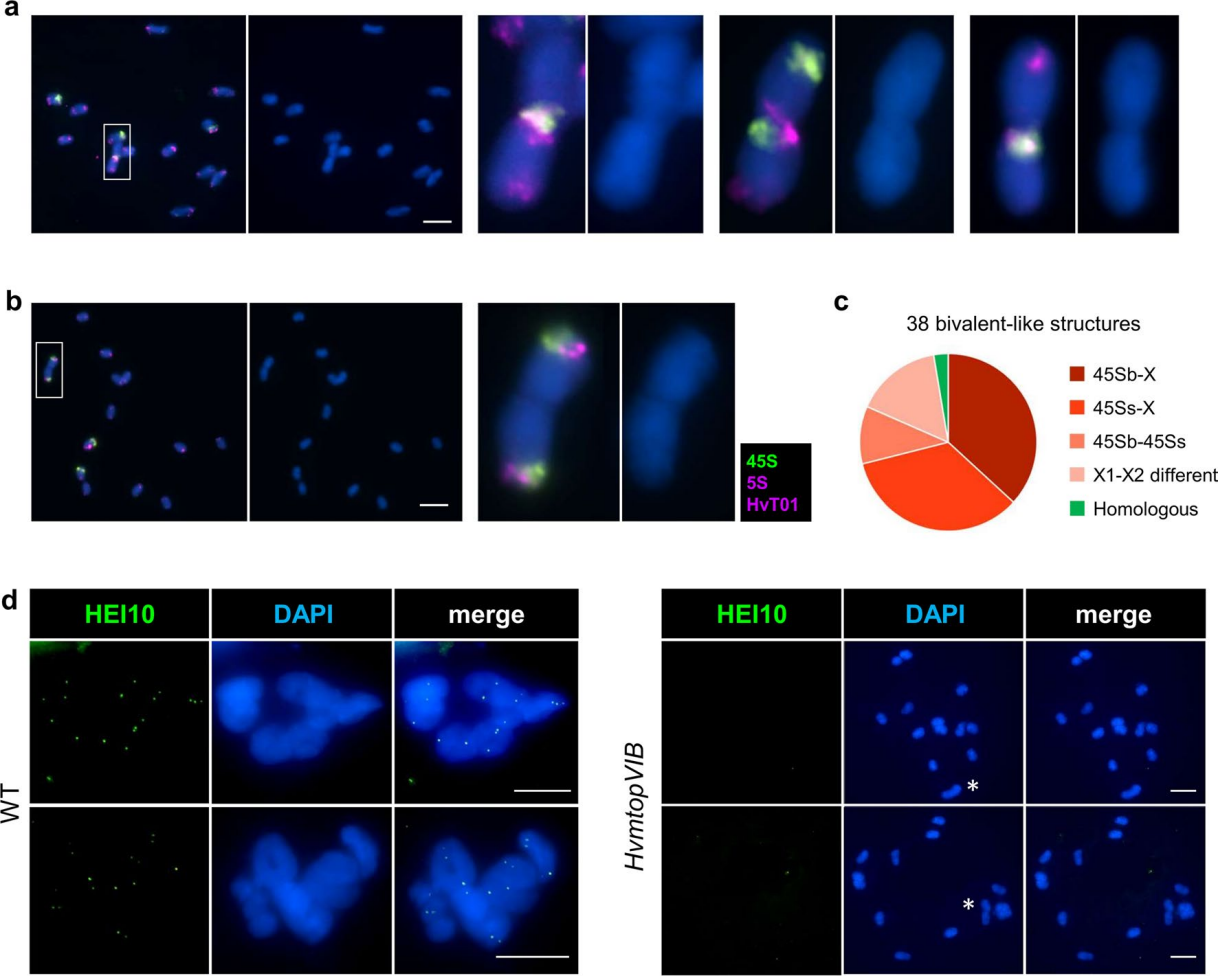
Bivalent-like structures in *HvmtopVIB* mutants are achiasmatic. **a**, **b** FISH with 45S (green) and 5S rDNA as well as HvT01 (purple) probes facilitate the identification of all homologous chromosome pairs. Among 38 bivalent-like structures, bivalent-like associations primarily occur between non-homologous chromosomes (a) and only in one case between homologous chromosomes (b). **c** Proportion of each type of bivalent-like structure (homologous, green; or non-homologous, red range) considering chromosomes involved (chromosome with large 45S rDNA signal (“45Sb”), small signal (“45Ss”) or no 45S signal (“X”)). **d** Immunolocalization of HEI10 in WT reveals numerous foci on diakinesis-metaphase I chromosomes. No HEI10 foci were detected on *HvmtopVIB* bivalent-like structures (asterisks) or univalents. DNA stained with DAPI and shown in blue. Bars represent 10 µm

Male meiotic chromosome analysis was performed to assess meiotic chromosome behavior in *HvmtopVIB* mutants. During WT meiosis (cv. Golden Promise) and in segregating WT plants of *HvmtopVIB* mutants (Fig. 2c), chromosomes appeared as thin threads during leptotene, gradually thickening as homologous chromosomes get physically connected, being completely synapsed during pachytene. Chromosome condensation led to seven bivalents connected via chiasmata (cytological visualization of COs), typically forming rings (representing at least one chiasma at each chromosome arm) and occasionally rods (at least one chiasma in one chromosome arm). During metaphase I, bivalents align at the equatorial plate, and homologous chromosomes segregate to opposite poles during anaphase I. Four haploid cells are found upon separation of chromatids during meiosis II, forming a tetrad that will give rise to male gametes. In *HvmtopVIB* (Fig. 2c), meiotic prophase I initially appeared similar to the WT. However, in *HvmtopVIB*, no cells showing thick chromosome threads representing pachytene were found. Moreover, during metaphase I, chromosomes appeared as 14 univalents, suggesting a failure to form chiasmata, which resulted in a lack of bivalent formation. During anaphase I, univalents segregated randomly to opposite poles resulting in unbalanced dyads and subsequently also in the formation of unbalanced tetrads and micronuclei. Notably, commonly also the precocious separation of sister chromatids was found during meiosis I. While in the WT, seven bivalents were invariably found, in *HvmtopVIB*, no bivalent formation was seen. However, structures reminiscent of a rod-like bivalent (1–3 per cell) were frequently observed in *HvmtopVIB* (Fig. 2d). To resolve whether infrequent bivalent formation occurred involving homologous chromosomes, FISH using HvT01 as well as 5S and 45S ribosomal DNA ribosomal DNA probes was performed (Fig. 3a–c). Among 38 studied bivalent-like structures, only one was formed by homologous chromosomes, a frequency which is even lower than the number expected by random associations. Notably, in 32 out of 38 cases, one of the two, or both, 45S rDNA-containing chromosomes participated in such bivalent-like structures, as previously observed even in the absence of recombination (Stronghill et al., 2010; Da Ines et al., 2012). However, not necessarily the 45S rDNA-containing chromosome arm was always involved. We also immunolocalized the class I CO marker HEI10 (Chelysheva et al., 2012; Wang et al., 2012) (Fig. 3d). While in the WT, 16.5 ± 1.3 HEI10 foci (range 14–20, n = 66) were found during diakinesis and metaphase I, in *HvmtopVIB* no HEI10 foci (n = 100) were found, including a complete lack of HEI10 foci at bivalent-like structures, suggesting the complete absence of ZMM-dependent class I CO. Hence, these bivalent-like structures likely represent random achiasmatic associations of chromosomes or chromosomes being nearby by chance.

### Absence of meiotic DSB and SC formation in *HvmtopVIB* mutants

**Fig. 4:**
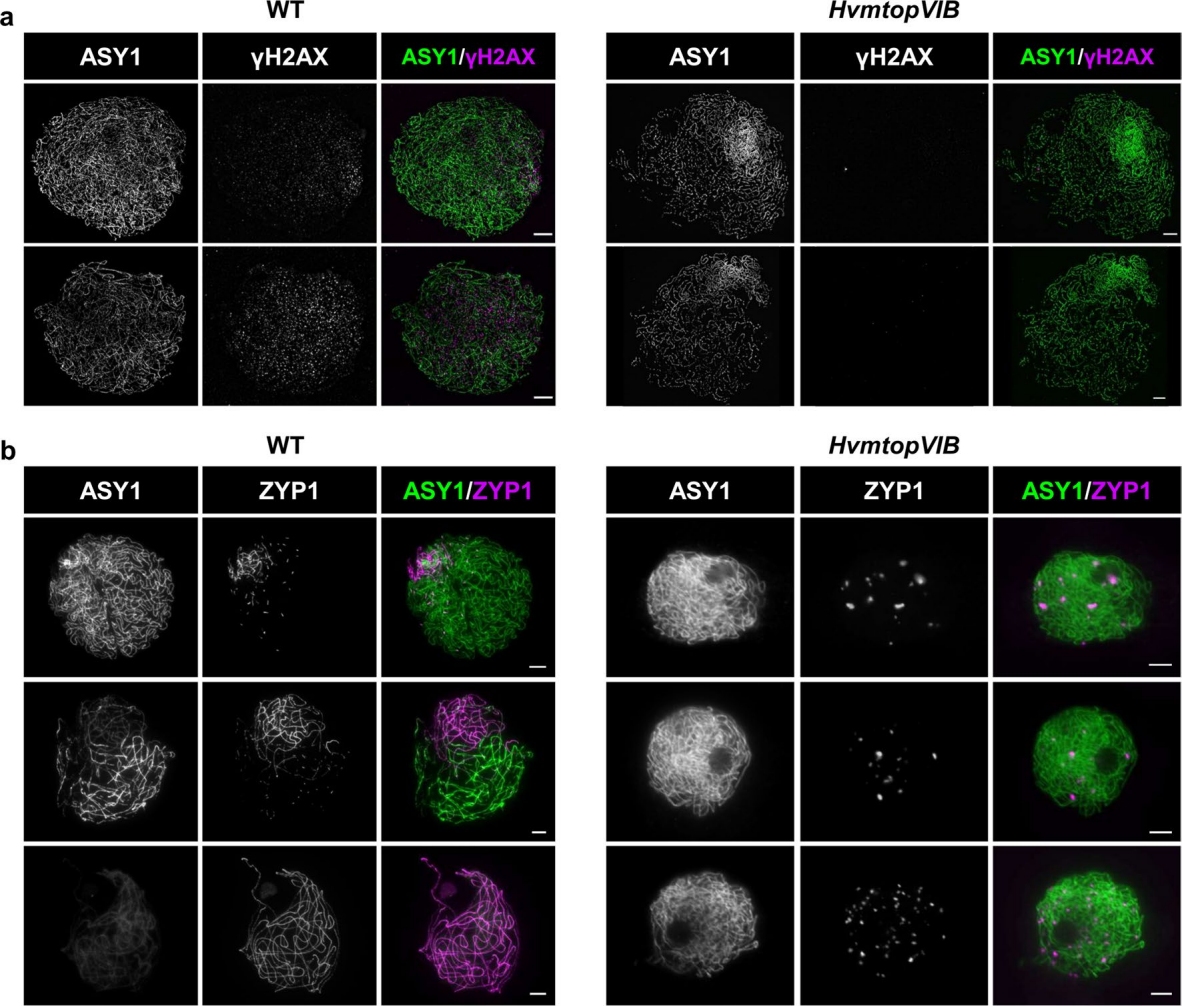
Absence of DSB and SC formation in *HvmtopVIB* mutants. **a** Immunolocalization of ASY1 and γH2AX visualized by 3D-SIM reveals numerous axis-associated γH2AX foci in WT but none in *HvmtopVIB*. **b** Immunolocalization of ASY1 and ZYP1 indicates initially polarized synapsis in WT spreading across the whole nucleus until all chromosome pairs are synapsed with ASY1 getting gradually depleted from ZYP1-positive synapsed regions (from top to bottom). In *HvmtopVIB*, only ZYP1 foci varying in number and size are present. No linear ZYP1 signals are found, indicating the absence of synapsis. Bars represent 5 µm

Rapid phosphorylation of histone H2AX around DSB sites is found and in diverse species including plants an antibody to detect the phosphorylated form of H2AX (γH2AX) is used to detect DSB sites (Rogaku et al., 1998, Paull et al., 2000; Mahadevaiah et al., 2001; Sanchez-Moran et al., 2007; Wu et al., 2015). Hence, we performed immunolocalization against γH2AX as a possible proxy for DSB formation in barley together with the axis-associated protein ASY1 (Caryl et al., 2000; Armstrong et al., 2002; Sanchez-Moran et al., 2007; Higgins et al., 2012) (Fig. 4a). In the WT, abundant γH2AX foci were detected during early zygotene (average 466.5 ± 113.2, range 352–675, n = 10), which is consistent with previous reports in barley (Higgins et al., 2012). In contrast, in *HvmtopVIB*, a strong decrease in γH2AX foci or complete absence was found during early prophase I (average 2.3 ± 2.8, range 0–8, n = 11), suggesting (near) absence of meiotic DSB formation. Thus, the absence of typical bivalent structures or ZMM-dependent CO formation is due to an abolished HR induction.

Simultaneous immunolocalization of ASY1 and ZYP1 (transverse filament protein of the SC; Higgins et al., 2005) was performed to explore whether SC formation occurs in *HvmtopVIB* (Fig. 4b). In the WT, ASY1 localizes to the meiotic chromosome axis during leptotene, and it becomes depleted from synapsed regions upon loading of ZYP1 (Wojtasz et al., 2009; Lambing et al., 2015). Hence, during zygotene-pachytene, the presence of ASY1 combined with the absence of ZYP1 marks un-synapsed chromosome regions, while the almost absence of ASY1 combined with the presence of ZYP1 marks synapsed chromosome regions. Moreover, a spatiotemporal asymmetry in meiotic progression was found, i.e., chromosome axis and SC formation are initially polarized to one area of the nucleus before gradually extending across the whole nucleus as previously described (Higgins et al., 2012). In *HvmtopVIB*, ASY1 localization was similar to WT during leptotene and zygotene including initial polarization before spreading throughout the nucleus. In the case of ZYP1, in *HvmtopVIB*, no typical linear signals were found. ZYP1 formed varying numbers of foci that did not elongate. Also, larger aggregates or polycomplexes that varied in number and size appeared. Moreover, in *HvmtopVIB* (n = 51) no sign of polarized localization of these ZYP1 foci or aggregates/polycomplexes was found. Thus, the absence of ZYP1 installation is consistent with abolished synapsis due to the lack of meiotic DSBs and consequently HR.

### Limited polyad formation and frequent bi-orientation of kinetochores in *HvmtopVIB* univalents during meiosis I

**Fig. 5:**
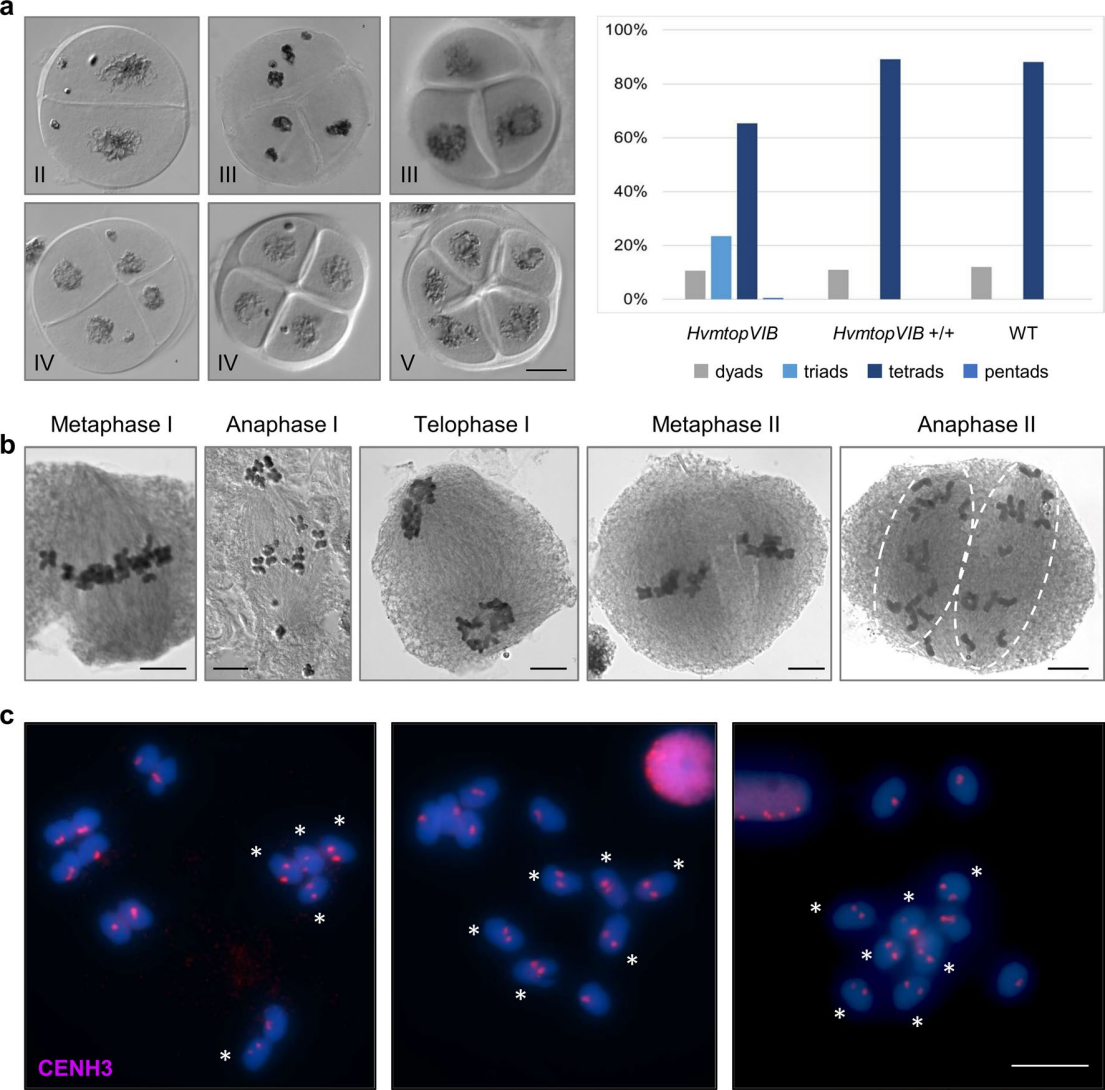
In *HvmtopVIB* mutants bipolar spindle formation including split sister centromeres in univalents and a limited frequency of polyads appears. **a** In *HvmtopVIB*, tetrads form predominantly; occasionally dyads similar to WT, sometimes triads, and only once a pentad. Acetocarmine-staining examples of a dyad (II), two triads (III), two tetrads (IV), and a pentad (V). **b** Acetocarmine-stained male meiotic cells reveal exclusively bipolar spindle formation in *HvmtopVIB*. From left to right: metaphase I, anaphase I, telophase I, metaphase II, anaphase II. **c** Immunolocalization of CENH3 (red) reveals split sister centromeres in univalents during diakinesis-metaphase I (two CENH3 signals per univalent, asterisks, vs. one signal that indicates fused sister centromeres). Chromosomes stained with DAPI and shown in blue. Bars represent 10 µm

MTOPVIB is critical for bipolar spindle formation during meiosis I in maize and rice (Xue et al., 2019; Jing et al., 2020). In *mtopVIB*, independent of meiotic DSB formation, multipolar spindles form, resulting in a high frequency of polyads instead of tetrads. Also, univalents preferentially have mono-oriented sister kinetochores during metaphase I. We asked whether sister kinetochore mono-orientation is prevalent also in *HvmtopVIB*, and polyads occur in high frequency. Surprisingly, in *HvmtopVIB* similar to the WT, we found only dyads after the first meiotic division, and predominantly tetrads (unbalanced and showing micronuclei formation) after the second meiotic division (Fig. 5a). Only one out of 179 spores in *HvmtopVIB* was a polyad, specifically a pentad. In accordance with this, spindles were consistently arranged in a bipolar fashion during the two meiotic divisions (83 cells) (Fig. 5b).

Both in WT and *HvmtopVIB*, dyads were found together with tetrads in the same preparations (in both backgrounds ~ 10% of all scored cells), likely representing asynchrony in meiotic progression among meiocytes within the same anthers as described for cv. Golden Promise grown at 20 °C (Schindfessel et al., 2021), which is thus unrelated to the *MTOPVIB* mutation.

Notably, in *HvmtopVIB* in addition to dyads and tetrads also triads were found (~ 23%), but never tripolar spindles or frequent polyads (Fig. 5a, b). A similar situation was found in other meiotic mutants forming two (dyads) or three (triads) nuclei unrelated to spindle alterations (e.g., *dmc1* in barley, where also pentads were found (Szurman-Zubrzycka et al., 2019), *pair2* in rice (Nonomura et al., 2004) or *mms21* in *Arabidopsis* (Liu et al., 2014)). Likely, due to defects in chromosome segregation in *HvmtopVIB* caused by the lack of recombination leading to unbalanced tetrads, cytokinesis that produces three or four (or very rarely five) microspores (often with micronuclei) occurs.

Using antibodies against the centromere-specific histone H3 variant CENH3, we quantified the number of split and fused CENH3 signals in *HvmtopVIB* univalents to quantify bi- and mono-orientation, respectively, of centromeres/kinetochores during meiosis I. All metaphase I cells contained at least 1 and up to 7 univalents with split centromeres (average 3 univalents/cell with split centromeres, N = 27 cells) (Fig. 5c), suggesting that bi-orientation of sister centromeres is frequent in univalents, and this could lead to the frequent observation of precocious sister separation found during anaphase I.

## Discussion

In this work, we have characterized *MTOPVIB* in barley. Mutant plants display an absence of DSB, SC, and CO formation resulting in the occurrence of univalents at metaphase I, unbalanced gametes, and sterility. Hence, HvMTOPVIB-dependent meiotic DSB formation is essential for meiotic recombination. Unlike other plants, MTOPVIB seems not to be required for bipolar meiotic spindle formation in barley.

### *HvMTOPVIB* is critical for meiotic DSB induction in barley

Disruption of *HvMTOPVIB* results in the absence of meiotic DSB formation and complete sterility, while vegetative growth and development are similar to WT. Reciprocal crosses suggest that both male and female meiosis are defective. No Y2H interaction was found between *HvSPO11-1* and *HvSPO11-2* while *HvMTOPVIB* interacts in yeast with both *HvSPO11-1* and *HvSPO11-2* similar to *Arabidopsis* and rice (Vrielynck et al., 2016; Xue et al., 2016). The interaction of *HvSPO11-1* or *HvSPO11-2* with *HvMTOPVIB* was disrupted in the case of *HvmtopVIB*, which lacks all four HvMTOPVIB domains conserved among flowering plants including the B4 motif part of the Transducer domain that mediates the interaction with SPO11-1 and SPO11-2 in plants (Vrielynck et al., 2016; Xue et al., 2016). Hence, similar to other plant species, also in barley, MTOPVIB may form a heterotetrameric complex with SPO11-1 and SPO11-2 constituting the meiotic TopoVI-like complex (core catalytic meiotic DSB induction complex) (Robert et al., 2016b). However, whether both *HvSPO11-1* and *HvSPO11-2* are indeed required for meiotic DSB formation and whether MTOPVIB indeed forms a heterotetrameric complex with SPO11-1 and SPO11-2 is unclear and needs further studies. Future studies are also needed to identify whether *HvSPO11-3* is required for meiotic DSB formation or whether *HvSPO11-3* is critical for somatic development and not required for meiotic DSB formation similar to *AtSPO11-3* (Hartung et al., 2002; Sugimoto-Shirasu et al., 2002; Yin et al., 2002).

### Absence of SC and CO formation in *HvmtopVIB *mutants

In *HvmtopVIB*, the absence of meiotic DSB formation results in severe meiotic defects that entail complete sterility. Meiotic chromosome axis formation during leptotene and zygotene, including an initial polarization of ASY1 before spreading throughout the nucleus was similar to WT in *HvmtopVIB*. However, synapsis was abolished in *HvmtopVIB*. Instead, ZYP1 formed varying numbers of foci that did not elongate. Hence, similar to most species including plants, also synapsis is meiotic DSB-dependent in barley.

Consistent with the absence of meiotic DSBs and accompanied meiotic homologous repair processes, we found no signs of meiotic CO formation. While in WT, seven bivalents are invariably found during metaphase I, in *HvmtopVIB*, only univalents were evident. The absence of bivalent formation resulted in unbalanced segregation of univalents into aneuploid gametes. Besides, precocious sister chromatid separation during meiosis I (see below) occurred. Notably, in some cells, bivalent-like structures were found at diakinesis/metaphase I. These structures lacked HEI10 foci and were randomly composed of either homologous or mostly non-homologous chromosomes. Due to this, and given the (near) absence of DSB (virtually no γH2AX foci) and SC formation (no ZYP1 polymerization), we consider these bivalent-like structures to be achiasmatic chromosome associations. Taken together, in barley, SC and CO formation requires meiotic DSB formation in an MTOPVIB-dependent manner.

### Bipolar spindle and limited polyad formation in *HvmtopVIB* mutants

Independent of its role in meiotic DSB formation, MTOPVIB is required for bipolar spindle formation during meiosis I in maize and rice (Xue et al., 2019; Jing et al., 2020). Moreover, regular spore formation is impaired in *mtopVIB* leading to primary polyads (up to octads) with a frequency of ~ 86% in rice (Xue et al., 2019), ~ 63% in maize (Jing et al., 2020), and ~ 70% in *A. thaliana* (Tang et al., 2017). In rice, at metaphase I, sister kinetochores of univalents were mono-oriented in *mtopVIB* but not in other DSB-defective mutant plants, where frequent bi-orientation of sister kinetochores was found. Hence, it was suggested that kinetochore bi-orientation in (at least some) univalents at metaphase I is essential for bipolar spindle organization and depends on MTOPVIB (Xue et al., 2019).

In *HvmtopVIB* meiotic bipolar spindles were found, and similar to the WT, the majority of tetrads were composed of four spores, that however, showed micronuclei and were genetically unbalanced. Thus, *MTOPVIB* is unlikely to be required for bipolar spindle formation in barley when univalents exist due to the absence of meiotic DSB formation. Furthermore, the frequent occurrence of bi-orientation of sister kinetochores in univalents during metaphase I in *HvmtopVIB* mutants, when compared with rice or maize *mtopVIB* univalents, where centromere mono-orientation was prevalent (Xue et al., 2019; Jing et al., 2020), suggests that bipolar spindle formation may indeed depend on sister kinetochore bi-orientation as proposed (Xue et al., 2019).

In rice, MTOPVIB interacts via its C-terminus with PRD2 (Xue et al., 2019) and in Arabidopsis *prd2* unequal bipolar or multipolar spindles form (Jiang et al., 2009), suggesting a possible functional interaction of MTOPVIB and PRD2. In *HvmtopVIB*, despite showing a DSB null phenotype and being the interactions with SPO11-1 and SPO11-2 disrupted in yeast, we cannot exclude that a truncated protein with residual function during metaphase I/kinetochore organization is formed. However, this seems rather unlikely since the predicted truncated *HvmtopVIB* lacks all conserved domains, including the domain required in rice for the interaction with PRD2 (Xue et al., 2019).

In any case, future studies of further DSB-defective barley mutants including additional mutant alleles of *mtopVIB* are required to address whether bi-orientation of sister kinetochores in univalents and meiotic bipolar spindle formation are indeed meiotic DSB-independent in barley.

Together, we showed that *HvMTOPVIB* is critical for meiotic DSB induction and that all accompanied downstream DSB-dependent processes including SC and CO formation are impaired in *HvmtopVIB* mutants. We assume that this mutant presents a valuable resource for future research on barley meiosis. Moreover, different from other species, *MTOPVIB* seems not to be required for meiotic bipolar spindle formation in barley.

## Author contribution statement

SS, MC, MAA, and CF performed experiments. VS performed super-resolution microscopy. IH, GH and JK designed and cloned transformation vectors and performed genetic engineering. SS, MC, and SH analyzed the data. SH acquired funding. SS, MC, and SH drafted the manuscript. All authors read and approved the final text.

## Supplementary Information

Below is the link to the electronic supplementary material.

Supplementary file1 (PDF 754 kb)
