# Supplementary material for: The meiotic topoisomerase VI B subunit (MTOPVIB) is essential for meiotic DNA double-strand break formation in barley (Hordeum vulgare L.)
Source: Plant Reprod. 2022 Jun 29;36(1):1–15. doi: 10.1007/s00497-022-00444-5 (PMC9957907; doi:10.1007/s00497-022-00444-5)
Supplement: Supplementary file 1 — Supplementary file1 (PDF 754 kb) [file 497_2022_444_MOESM1_ESM.pdf]

The meiotic topoisomerase VI B subunit (MTOPVIB) is essential for meiotic DNA double-strand break formation in barley (*Hordeum vulgare* L.)

Journal name: Plant reproduction

Stefan Steckenborn*, Maria Cuacos*, Mohammad A. Ayoub, Chao Feng, Veit Schubert, Iris Hoffie, Götz Hensel, Jochen Kumlehn, Stefan Heckmann

*authors contributed equally

Leibniz Institute of Plant Genetics and Crop Plant Research (IPK) OT Gatersleben, Corrensstrasse 3, 06466 Seeland, Germany

Corresponding author: Stefan Heckmann, heckmann@ipk-gatersleben.de

Name	Sequence (5' → 3')
**Sequencing of *HvMTOPVIB* coding sequence**	
HvMTOPVIBcdsFWR	ATGCCGTCCCCGTCC
HvMTOPVIBcdsrREV	GAAATCAAAAATCATATCCTCGTC
**Yeast Two Hybrid assays**	
HvSPO11-1y2hFWR	agattacgctcatATGGCGGGGAGGG
HvSPO11-1y2hREV	cgagctcgatggatccCTATATATACCTACCCAGTTTGATC
HvSPO11-2y2hFWR	agattacgctcatATGGCGATGGCGGAGG
HvSPO11-2y2hREV	cgagctcgatggatccTCAAATGTAATCACCCTGCAC
HvMTOPVIBy2hFWR	agattacgctcatATGCCGTCCCCGTCC
HvMTOPVIBy2hREV	cgagctcgatggatccctaGAAATCAAAAATCATATCCTCGTC
**Cloning of target-specific sequences for Cas9-triggered mutagenesis**	
GH-MTOPVIB-g1-FWR	CTTGAGCTTCCGGTGGGGGGAGG
GH-MTOPVIB-g1-rev	AAACCCTCCCCCCACCGGAAGCT
GH-MTOPVIB-g2-for	CTTGGATGTCGGAGTCGCAGTGC
GH-MTOPVIB-g2-rev	AAACGCACTGCGACTCCGACATC
GH-MTOPVIB-g3-for	CTTGACTTCATATTATGGCTGGT
GH-MTOPVIB-g3-rev	AAACACCAGCCATAATATGAAGT
**Mutation identification and *HvmtopVIB* genotyping**	
HvMTOPVIBmutFWR1	ACCACAATTCACAAACCCC
HvMTOPVIBmutREV1	CTGACACTGTGACTAACTAGC
HvMTOPVIBmutFWR3	GGGTTCTTGTCTTCCTCCGT
HvMTOPVIBmutREV3	CGGGATTCTAATATTGACAGGG
**Identification of transgene-free individuals**	
Cas9-genotyping-FWR	TTTAGCCCTGCCTTCATACG
Cas9-genotyping-REV	TTAATCATGTGGGCCAGAGC
Hygro-genotyping-FWR	AGCGAGAGCCTGACCTATT
Hygro-genotyping-REV	GTCCGAATGGGCCGAAC

Supplementary Table 1: List of primers used during this study.
